# Feasibility and acceptability of a proposed trial of acupuncture as an adjunct to lifestyle interventions for weight loss in Polycystic Ovary Syndrome: a qualitative study

**DOI:** 10.1186/s12906-018-2358-7

**Published:** 2018-11-08

**Authors:** Carolyn Ee, Caroline Smith, Michael Costello, Freya MacMillan, Lisa Moran, Brandi Baylock, Helena Teede

**Affiliations:** 1 NICM Health Research Institute, Western Sydney University, Locked Bag 1797, Penrith, NSW 2751 Australia; 2 School of Women’s and Children’s Health, UNSW, Royal Hospital for Women, Barker St, Randwick, NSW 2013 Australia; 3 School of Science and Health and the Translational Health Research Institute, Western Sydney University, Locked Bag 1797, Penrith, NSW 2751 Australia; 4 Monash Centre for Health Research and Implementation – MCHRI, School of Public Health and Preventive Medicine, Monash University, in partnership with Monash Health, Level 1, 43-51 Kanooka Grove, Clayton, Vic 3168 Australia; 5 Western Sydney University, Locked Bag 1797, Penrith, NSW 2751 Australia

**Keywords:** Acupuncture, Polycystic ovary syndrome, Lifestyle interventions, Telephone-based health coaching, Randomised controlled trials, Obesity, Qualitative, Sham acupuncture

## Abstract

**Background:**

Polycystic Ovary Syndrome (PCOS) is a common female reproductive disorder with multiple manifestations. Weight management is a key therapeutic goal. Acupuncture is a potential adjunctive weight loss treatment in non-PCOS populations. We aimed to engage patients in co-design and assess the feasibility and acceptability of methods for a randomised controlled trial (RCT) on acupuncture and telephone-based health coaching for weight management in overweight or obese women with PCOS using qualitative methods.

**Methods:**

We recruited women who had PCOS and were aged 18–45 years and with a body mass index of 25 kg/m^2^ and over, using social media. Two face-to-face focus group meetings and three semi-structured telephone interviews were conducted (*n* = 10). We analysed data using thematic analysis and aimed to compare and contrast motivations for joining the trial between women who were actively trying to conceive (*n* = 7) and not trying to conceive (*n* = 3). Attitudes to, knowledge and experiences of acupuncture; perceptions and attitudes towards the interventions in the RCT (real acupuncture, sham acupuncture and telephone-based health coaching); the outcomes of importance; and barriers and facilitators to successful trial recruitment and retention were collected.

**Results:**

Women were both acupuncture-naive and acupuncture-experienced. Overall, attitudes towards acupuncture were positive, and the trial design was acceptable with appointment flexibility requested. Ideal enrolment time, if women were trying to conceive, was six months prior to conception. Women supported three-month intervention and the use of sham acupuncture as a control. Financial incentives were not believed to be necessary, and women spoke of altruistic intentions in enrolling for such a trial. Women who were trying to conceive voiced a need for support from their family, health coaches, and peers. The telephone-based health coaching offered welcome support and accountability, noted as possible facilitators of weight loss.

**Conclusions:**

Our findings show that acupuncture is a likely acceptable adjunct to lifestyle interventions for weight loss in PCOS, and that a sham-controlled trial is feasible and acceptable to PCOS women. Further research is required in order to evaluate the efficacy of acupuncture together with lifestyle for weight management in PCOS.

**Electronic supplementary material:**

The online version of this article (10.1186/s12906-018-2358-7) contains supplementary material, which is available to authorized users.

## Background

Polycystic Ovary Syndrome (PCOS) is the most common endocrine disorder amongst reproductive aged women [1], with a prevalence of up to 17.8% amongst Australian women [2]. PCOS is a complex disorder, with reproductive, metabolic and psychological manifestations [3]. Women with PCOS are more likely to be obese/overweight than age-matched controls [4], and excess weight worsens the features of PCOS [5]. Weight management is a key goal in PCOS and lifestyle management targeted at weight loss are first-line recommendations in overweight or obese women [6]. However, adherence to lifestyle interventions is generally low, with dropout rates of up to 62% reported in randomised controlled trials [7]. Further, suboptimal lifestyle behaviours have been reported amongst women with PCOS which could contribute to difficulties with weight management. A large longitudinal study reported that women with PCOS consumed 250 more kilojoules and had an additional half hour sitting time per day compared with women without PCOS [8]. One cross-sectional study reported that less than half of respondents with PCOS were physically active, compared with two thirds of respondents without PCOS [9] . Achieving adequate weight loss remains a significant challenge for many women with PCOS [10].

Women with PCOS are frequent users of complementary therapies, and up to a third will use acupuncture [11]. In non-PCOS populations, acupuncture is more efficacious than sham acupuncture for weight loss, with mean differences in body weight and body mass index (BMI) of 1.58 kg [12] to 4.4 kg [13], and 0.6 kg/m^2^ [12] to 2.79 kg/m^2^ [14] respectively (3.5–5% decrease in weight from baseline) [12–15]. There are several putative mechanisms including modulation of vagal tone [13, 15–27]. Acupuncture may represent a low-risk [28] non-pharmacological adjunct to lifestyle interventions for weight loss in PCOS, but rigorous sham-controlled trials evaluating acupuncture are needed to confirm these preliminary findings in women with PCOS. However, many RCTs fail due to the inability to recruit to target [29], including a recent trial comparing acupuncture and sham for live birth rate with In-Vitro Fertilisation [30]. To this end, the UK Medical Research Council recommends assessment of feasibility prior to full evaluation of complex interventions [31]. This ensures that money spent on expensive trials is not wasted due to recruitment and retention failures. Qualitative research involving community stakeholders can assist with exploring acceptability and feasibility of trial procedures, and ensures that proposed interventions are translatable and more likely to be sustained in real-world settings [32].

The aim of this study was to conduct a qualitative study to assess the feasibility and acceptability of a method for a sham-controlled RCT on acupuncture and evidence-based lifestyle interventions (telephone-based health coaching) [33] for weight management in overweight or obese women with PCOS.

## Methods

Our research questions were:

- What are women’s perceptions, knowledge, experiences and attitudes towards the use of acupuncture for weight management in PCOS?
- To what extent is the proposed method for an RCT on acupuncture as an adjunct to lifestyle interventions (telephone-based health coaching) for weight management in PCOS acceptable to women?
- What do women perceive as the barriers to and facilitators of a successful RCT on acupuncture as an adjunct to lifestyle interventions for weight management in PCOS (with regards to successful recruitment, minimising attrition rates, and optimising compliance and adherence), how do the barriers and facilitators differ by pregnancy intention, and how can these barriers be overcome?

### Qualitative study design

This qualitative study used a combination of two face-to-face focus focus groups, and three semi-structured individual telephone interviews for women unable to attend focus groups. Ethics approval was obtained from the Western Sydney University Human Research Ethics Committee (H11935/28 Nov 2016) and all participants provided written informed consent.

### Participants

We invited women from the community to participate through social media, using both paid advertisements and unpaid posts on Facebook pages or online newsletters relating to PCOS or women’s health. Participants were eligible for inclusion if they self-reported having been diagnosed with PCOS and self-reported meeting the 2003 Rotterdam Criteria [16]; self reported BMI > 25 kg/m^2^; had no other endocrine disorders; were not currently pregnant; were at least six weeks post-partum if recently pregnant; were fluent in English, and able to attend for a focus group in the Western Sydney area.

Purposive sampling was used to target two populations: (1) Women seeking pregnancy who have or have not been diagnosed with infertility (Trying To Conceive/TTC group), and (2) Women not seeking pregnancy (not Trying To Conceive/not TTC group). We hypothesised that these two populations may be motivated differently in terms of weight management, and this may impact on acceptability of the proposed trial procedures. One focus group of 6–8 participants was a priori planned for each of the two populations. Focus group sizes of 6–12 participants are suggested in the literature [34] however we chose a smaller group size as we aimed to explore potentially sensitive issues in depth and our previous experience indicates this is best conducted in a more intimate group setting with fewer participants.

### Data collection and analysis

An experienced research officer (BB) organised and moderated the focus groups while another investigator (CE) observed and took field notes. A Health Promotion undergraduate student experienced in interview techniques (LI) conducted the telephone interviews. Focus group discussions and telephone interviews were also audio-recorded and professionally transcribed verbatim.

Basic demographic data, including age, self-reported BMI, ethnicity, educational level, income, and marital status, as well as complementary therapy use, were collected from participants, using a paper-based survey for the focus groups and the same questions on an online survey for women who provided a telephone interview. Women were given verbal and written information about the proposed RCT during the focus groups /telephone interviews/ (see Table 1).

**Table 1:** Details of the proposed RCT methodology which were provided to participants

Design	Two group parallel design RCT comparing real acupuncture with sham with both groups also receiving lifestyle coaching. Women will have a 50% chance of being allocated to receive either real or sham acupuncture.
Population	Women with PCOS who are overweight or obese
Interventions	All women will receive lifestyle coaching over the telephone (ten 15-min phone calls over 6 months), and will set their own diet and exercise goals, with an aim to achieve the national guideline recommendations for diet [52] and exercise [53]. The intervention group will also receive 12 treatments of real acupuncture over 12 weeks (45 min sessions delivered twice weekly for the first 4 weeks and then fortnightly for 4 sessions).
Control	As well as lifestyle coaching, the control group will also receive 12 treatments of sham acupuncture over 12 weeks (45 min sessions delivered twice weekly for the first 4 weeks and then fortnightly for 4 sessions). Sham needling (referred to as “placebo acupuncture” in the participant information) is when needles are not inserted in the way they are done usually, and is therefore believed to be less active than real acupuncture.
Outcomes	Primary outcome: weight loss. Secondary outcomes: two hour Oral Glucose Tolerance Test and other blood tests, surveys on different aspects of health
Blinding	Women will be blinded.

We developed a question guide for the purposes of this study. Details can be found in Additional file 2. Briefly, we asked about:

- past weight loss attempts
- acupuncture: previous experiences and knowledge, perceptions of advantages and disadvantages, perceptions and attitudes of its potential role as an adjunctive weight loss treatment
- interventions of real acupuncture and telephone-based health coaching in the RCT: perceptions and attitudes towards telephone-based health coaching and acupuncture in terms of appealability to the target population, acceptability in terms of time commitment and adverse event profile. We explored these both separately as individual interventions (acupuncture, telephone-based health coaching) as well as asking about the combination of the two interventions.
- the control method of sham acupuncture in the RCT: perceptions on how this might impact on recruitment and attrition, whether other attention control methods may be more acceptable such as massage or progressive muscle relaxation
- the outcomes of the RCT that are important to them as end-users, the acceptability of the proposed outcomes in the RCT
- feasibility of the RCT: perceptions of barriers to and facilitators of successful recruitment and retention for this trial, exploring issues such as incentives and recruitment sources.

The method of constant comparison [35] was used in a thematic analysis of the data. Members of the research (BB, CE, FM) team independently coded a subset of the transcripts before participating in a data analysis workshop in which a set of codes were generated and agreed upon. It was agreed that these codes captured the women’s attitudes regarding the idea of the RCT. This coding scheme was then refined to include a number of further codes and subcodes for the two groups of women seeking pregnancy and those not seeking pregnancy. After a second reading of these codes and subcodes, the emergent codes were used to code the complete dataset. Finally, the subcodes were used to compare groups within the higher order themes. The robustness of the analysis was assessed by cross-checking the coding schema of a subset of transcripts among the researchers, resulting in high matching of these analyses. In all, 20% of data was cross-checked. Microsoft Excel 2016 version 15.40 was used to manage the data. Women were given pseudonyms to maintain confidentiality.

## Results

We recruited women over a 3 month time period from December 2016 until the end of March 2017. The majority (123/194 or 65%) of potential participants responded to promotion via Facebook paid advertisements; the remainder responded to unpaid posts on Facebook pages or groups, online newsletters, and word of mouth.

**Fig. 1:**
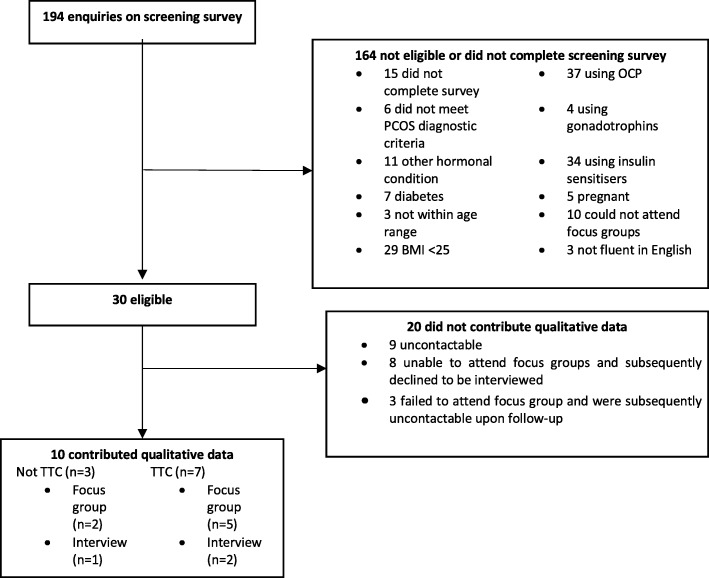
Flow of participants through the study. TTC = Trying To Conceive

Figure 1 describes the flow of participants through the study.

Overall, ten participants provided qualitative data. Of the seven participants who were trying to conceive, five reported difficulty in conceiving (no pregnancies despite TTC for at least 12 months) and 2 reported having tried to conceive for less than 12 months. The mean duration of the focus groups were 110 min (range 104 to 117 min) compared to the mean duration of individualized telephone interviews being 21 min (range: 17 to 27 min). We had initially proposed to recruit 12–16 participants in each of the two focus groups (TTC and not TTC). As stated earlier, one-to-one telephone interviews were offered for convenience where necessary. Although our target sample size was not met, saturation in the discussions was met – no new major themes arose towards the completion of data collection.

Table 2 shows the demographic details of participants. Nine women completed the demographic survey. One woman, who provided a telephone interview, did not complete the demographic survey despite repeated reminders. Almost half of the women had used a complementary therapy in the previous 12 months, including two who had used acupuncture. The majority of women were of European ancestry, married, and had at least a Bachelor degree qualification. One third of women were of Asian origin.

**Table 2:** Participant demographic details

	Mean (SD) or number of participants(%)
Pregnancy intention	
Trying to conceive (TTC), no difficulty	2 (20%)
Trying to conceive (TTC), difficulty	5 (50%)
Not trying to conceive	3 (30%)
Age in years (SD), all participants	36.1 (7.24)
Age in years (SD), not TTC (*n* = 2)	43.5 (2.12)
Age in year (SD)s, TTC (*n* = 7)	34.0 (6.76)
Missing (*n* = 1)	
Use of complementary therapies in previous 12 months	
Yes	4 (40%)
No	5 (50%)
Missing	1 (10%)
Types of complementary therapy used (*n* = 4)	Vitamins (2)
	Acupuncture (2)
	Chinese herbs (1)
	Vitamin D (1)
	Fish oil (1)
	Chiropractic (1)
Marital status	
Married	5 (50%)
De facto^a^	2 (20%)
Single	2 (20%)
Missing	1 (10%)
Employment	
Employed full time	9 (90%)
Missing	1 (10%)
Ethnicity/race	
European	5 (50%)
Asian	3 (30%)
Oceanic	1 (10%)
Missing	1 (10%)
Highest educational achievement	
High school - Year 10	1 (10%)
TAFE or vocational college	2 (20%)
Bachelor Degree	5 (50%)
Postgraduate	1 (10%)
Missing	1 (10%)
Gross weekly income (annual income)	
$600–799 ($31,200 - 41,599)	1 (10%)
$1000–1249 ($52,000 - 64,999)	3 (30%)
$1500–1999 ($78,000 - 103,999)	5 (50%)
Missing	1 (10%)
BMI, all participants	36.38 (SD 7.8)
BMI, not TTC (*n* = 2)	31.90 (SD 6.45)
BMI, TTC (*n* = 7)	37.66 (SD 8.1)

One woman did not return demographic data

*BMI* Body Mass Index, *SD* Standard deviation, *TAFE* Technical And Further Education, *TTC* Trying To Conceive

^a^also known as common-law marriage or cohabitation

Two major themes emerged: (i) Acupuncture (advantages, knowledge of, and disadvantages); (ii) Trial design (general impressions, acceptability of the interventions, other suggestions, other aspects of trial design, and feasibility). We refer to women using their given pseudonyms, and also by pregnancy intention (TTC/not TTC). Additional quotes not presented in this manuscript can be found in Additional file 1.

### Acupuncture: “If it really helped why not”

#### Advantages

Overall, women were open considering acupuncture as an additional weight loss intervention. Advantages were that it was a “holistic”, non-pharmacological intervention and may help with other aspects of PCOS such as stress, menstrual cycles and fertility. Women felt that if it was found to be helpful, they would consider using it. Harriet was influenced by her trust in Chinese medicine. Others, like Keira, had not tried it before, but were willing to give it a try, even though the experiences of others had been equivocal.

However, the willingness to try acupuncture was seen within the context of a lifetime of trying multiple interventions for PCOS and weight loss, as Jessica (TTC) said:

*“I’m sure we’ve spent lots of money trying other things and everything else.”*

### Experiences with and knowledge of acupuncture

There was a mix of women who were acupuncture-naive and acupuncture-experienced. Women who were acupuncture-experienced described positive experiences, including relief of chronic conditions such as migraine and dysmenorrhoea, a feeling of relaxation, and stress management. Sometimes, the benefits of acupuncture treatment less well-defined, as in the case of Zoe (TTC) who reported that it seemed to brighten up her skin (“*I don’t know, I felt shiny afterwards*”) and Deanna (TTC) who reported that she “*loved going*” even though acupuncture had not had any impact on her ability to fall pregnant.

Women who were acupuncture-naive had varying levels of knowledge about the treatment, but were willing to give it a try. Annabelle (not TTC) knew it could be used for pain and that it involved “*the little needles*”. Jessica (TTC) thought the needles were inserted to affect some kind of “*release*”. Grace (TTC) thought it was “*supposed to help with a lot of different things*”, while Keira had a friend who was trying acupuncture for fertility.

### Disadvantages

The main disadvantages of acupuncture were financial cost, time commitment and the fact that it involved needles. Jessica (TTC), who was acupuncture-naive, confessed she was “*terrified of needles”.* Georgia (TTC) thought she had “*a bit of needle phobia*” but said “*once I realised that it really didn’t hurt or anything then it was fine.”*

For some, the acupuncture experience had some negative aspects, which were complex and intertwined. Chloe (TTC) spoke of difficulty getting to the acupuncture appointment, and a sense of failure.

*“The problem is to get to her - to get there I’d have to leave work at a certain time and I have to bout through all this traffic just to get there. So, I’d often turn up anxious and she’d feel my pulse and she’d be like you’re quite anxious. Often, I would try because I’m failing at losing weight, I’m failing at eating right, I’m failing now at having a kid and now I’m failing at trying to calm down even though I feel calm but obviously I’m not” -* Chloe, TTC.

Annabelle (not TTC) who was acupuncture-naive, also spoke about the lack of awareness and acceptance in the medical profession, and the somewhat negative view in Western culture that acupuncture was “*something that Asian people went to go and do. But Western societies looked at it and went, oh I don’t know about that one ”* She also spoke of the difficulty in choosing a reliable practitioner:

*“If you don’t know someone who goes then you’re not necessarily just going to turn up to anybody because you don’t really know what they’re doing. You don’t understand it enough just to go there.”*

Chloe (TTC) was not sure whether it was the treatment itself that helped her or if it was “*that I was doing something and I was giving myself an hour out of the day for the whole week for me, when I probably could have taken a walk for an hour and that could have been giving me the same satisfaction.”*

### Trial design: “It’s like a test on yourself”

#### General impressions

Overall, women felt that the design of the clinical trial was appropriate, and was something that they would consider credible. In particular, they responded positively to the duration of the trial (three months) and the fact that it was combined with lifestyle interventions. There was consensus that three months was required in order to implement and sustain new habits and to see a difference. Anything less would not be seen as credible or appropriate.

Women also felt that the overall trial design was feasible, and not too onerous. Annabelle (not TTC) described the trial as “*easy*” and “*flexible*”. Others, like Georgia (not TTC) had initial concerns about issues like location of the acupuncturist and the ability to attend outside of working hours; if these issues could be addressed, then trial participation would be feasible.

### Acceptability of interventions

#### Acupuncture frequency

Several women in the TTC group felt that twice-weekly visits would not be sustainable or realistic, while women in the group not TTC felt they could commit to twice-weekly visits if it was convenient to them. Convenience meant the ability to attend after working hours (in the evenings or on weekends) and at a location that was close to home or work.

#### Telephone-based health coaching

In general, women in both groups liked the idea of telephone coaching. It offered them accountability, which they felt was an important part of successful weight loss, and would be easier to fit into their busy lives compared to face-to-face appointments. However, women in the TTC group saw the lifestyle coaching as a possible means of support instead of simply coaching. Some, like Grace, thought that face-to-face appointments would be “*that bit more personal”* while Sarah wanted “*someone to keep in touch with”*. Chloe felt it would be important to be supported if she was not having a successful week:

*“I think the hardest thing is being aware and not being so hard on yourself if they do call and you haven’t probably been as effective that week, but they’re there to support you through that. I think the key would be the support. Not so much being so accountable but are they there for support and how would you use them. We might all use them differently”* ***-*** Chloe, TTC.

### Other suggestions

#### PCOS specific coaching

Having a coach who had had knowledge of PCOS was seen as important by women in the TTC group. Chloe spoke of an experience of calling a similar service and being laughed at when she mentioned she had insulin resistance, which left her feeling offended and embarrassed. The need for PCOS-specific advice extended beyond the coach having medical knowledge of the condition; it was important for women to have a supportive and understanding coach who grasped the impact of this chronic and treatment-resistant condition, and who could provide an appropriate level of support.

*“Yeah, even doctors don’t know a lot about it in my own experience. Especially in terms of weight management because it is quite tricky to lose weight with PCOS I’ve found. Having someone that actually knows more about that would be good” -* Sarah, TTC.

#### PCOS support group

Women in the TTC group expressed a strong need for peer support from other women with PCOS, and a preference for this support to be available face-to-face. It was suggested that this could be incorporated into the trial as an additional source of accountability and encouragement. There was consensus that frequent meetings were not necessary; a monthly meeting would be ideal. Women were seeking an experience of a shared identity and of mutual understanding, which would increase their motivation to complete the trial.

*“Yeah, probably accountability to other women who are going through the same thing would be a big thing as well.” -* Deanna, TTC.

#### An individualised approach

Women TTC also expressed the need for flexibility within the trial and an opportunity to have an individualised approach if they were not seeing results. There was a sense that each woman was unique, and that the approach should not be a “one size fits all”. Women had a variety of suggestions including additional support at a certain stage, some face-to-face sessions if needed with a nutritionist, and an increase in frequency of support.

*“I would [want an individualised approach] because I’m just thinking along the lines of going to those groups and having other people experience losses and things like that and me sitting there and not having anything [inaudible], because even in a group of people all with PCOS everybody’s different” -* unidentified female, TTC Focus group.

### Other aspects of trial design

Women were supportive of all other aspects of trial design, such as the outcome collection and sham group. Most women agreed that it would not matter to them if they were allocated to a sham group, and some women thought the process of receiving sham would be an interesting exercise in itself. There was an understanding that this was important in order for the study to be scientifically valid, and a willingness to put aside personal need in order to contribute to PCOS research.

*“We’re participating in a trial so we are aware and the reason why we’re participating in this trial is because - not because we want to get the real needle; we want to know the answers. We want to know what our future - what - we want to know more about PCOS. So, me, I personally - if I got the sham needle or not I feel like I’m contributing to…” -* Chloe, TTC.

However, it was suggested that offering a course of real acupuncture treatment to women in the sham group at the end of the study would be additional motivation to join the trial.

Attending for a 2-h OGTT was not seen as onerous as many had already attended for one in the past. Neither was offering a financial incentive seen as necessary. All women agreed that they would volunteer for the trial regardless of any financial reimbursement (though reimbursement for travel was seen as attractive).

*“But me personally, I’d just do it for the possibility that it could help. Do you know what I mean?” -* Grace, TTC.

### Feasibility

#### Barriers to participation

Women in both groups (TTC and not TTC) generally spoke of the same potential barriers to participation in the trial. Consistently, the inconvenience of attending acupuncture treatments was seen as a major potential barrier. This involved a time commitment and fitting treatments around an already busy schedule. Fear of needles was also spoken of in both groups as a possible barrier for some women.

Women in the not TTC group felt that support from one’s family was important. Women in the TTC group spoke of non-PCOS specific interventions being a potential barrier, as well as any interference with reproductive plans. These women felt that it would not be ideal to postpone or interrupt assisted reproductive plans, nor would it be acceptable to “*stuff this trial up”* if they had to decide to withdraw from the trial due to starting a new In Vitro Fertilisation (IVF) cycle. Indeed, a discussion about IVF affecting the trial was couched in terms of not wishing to adversely affect trial results if the participant started IVF or had to withdraw. It was suggested that an ideal time to enrol in the trial would be in the 6 months before starting to try to conceive.

*“I’d be worried if you’re starting this clinical trial but then you get - you’re then also put into a - you need to start IVF ASAP because of your age how that would affect the trial. I mean I’ve heard lots of studies as well that acupuncture during IVF is great for your stress levels more so than anything. So, would anybody be making the trial a risk if they were going to do IVF or something” -* unidentified female, TTC.

#### Suggestions for recruitment

Women in both groups had a variety of suggestions on how to recruit for the trial. Most women mentioned social media as an effective way of recruiting. As well as this, advertising in health professionals’ clinic rooms was suggested, such as in GP clinics, gynaecology/fertility specialist rooms, and acupuncturists.

*“Because when I go to the gynaecologist, I always read - they have this big noticeboard thing, so I’m always looking at stuff while I’m waiting”* - Grace, TTC.

#### Motivation to join the trial, and outcomes of importance

Not surprisingly, women in the TTC group spoke of the chance to boost fertility and/or have a healthy pregnancy being a motivation to join the trial. All women in the TTC group agreed that fertility was their top priority in terms of the outcome of interest, regardless of whether or not they had experienced any difficulty getting pregnant. Weight loss was seen in the context of improving chances of falling pregnant and having a healthy pregnancy.

For women not TTC, weight loss was a major motivator for enrolling. Even more of a motivator was the thought of weight maintenance and an end to the rollercoaster of weight loss and weight gain. Women in this group also spoke of their concerns about chronic disease in the future such as heart disease.

*“I want to do it. I really want to lose weight. I don’t want to be yoyo again up and down. As I say I need commitment and I need someone pushing me...Yeah, that’s what I want for the long term. Short term, I’ve tried too many short term” -* Harriet, not TTC.

## Discussion

This novel study evaluated the feasibility of a clinical trial on acupuncture integrated with lifestyle for weight loss in overweight or obese women with PCOS.

### Acupuncture experience and trial acceptability

Overall, the women in our study found trial design to be acceptable and feasible, had a positive view of acupuncture, and agreed it would appeal to many women with PCOS. Perceived advantages of acupuncture include a non-pharmacological approach and potential benefits to other aspects of health. Similarly, Australian women with PCOS identified the ability to treat more than one clinical problem as an advantage of using complementary therapies in a national survey [11]. There was an openness to trying acupuncture as a potential weight loss intervention and to contribute to research around its effectiveness.

Women supported the three-month duration of treatment, as many believed that weight loss can only be sustained if an intervention lasts at least 3 months. Gokee-LaRose et al. also reported that 10 weeks was believed to be a sufficient length of time for an intervention amongst the majority of young adults in a trial on behavioural self-modification for weight loss [36].

Many women supported the use of a sham control. This is consistent with previous research indicating that in general, participants of complementary therapy trials understand the need for RCTs and the value of the scientific method [37, 38]. Knowledge of placeboes may be relatively high amongst the general public, with a UK-based survey conducted on participants recruited from the community indicating that 68% of respondents had heard of or read about placeboes [39]. Although our findings may reflect our well-educated cohort, it is consistent with the real-world finding that the majority of complementary therapy users tend to be more well-educated women [40], and may signify ease of translation of our research evidence to end-users.

Some perceived disadvantages of having acupuncture include the need to find a reliable and credible practitioner, and a fear of needles. We are addressing these concerns by choosing trial acupuncturists with a minimum 5 years clinical experience, and will communicate this to future RCT participants. We will explain in the Participant Information and Consent Form that acupuncture needles cause significantly less discomfort than needles in other medical settings (such as injections and phlebotomy) because they are much finer and non-bevelled.

Acupuncture may be a beneficial adjunct to lifestyle interventions through its putative actions on the vagal nerve, resulting in appetite suppression [16, 17]. This effect may be mediated through the obesity-related peripheral peptides leptin and ghrelin [13, 15, 18]. Acupuncture may also improve insulin sensitivity [19–24] and may further impact on weight by reducing anxiety [25–27]. It is plausible that acupuncture may be a novel adjunctive treatment for weight loss that can both optimise behavioural change through reducing anxiety and appetite, as well as impacting on physiological markers such as leptin, ghrelin and insulin. Our findings suggest it may be a feasible adjunctive intervention for weight loss, and further clinical research is warranted.

### Barriers and facilitators to participation

The time and inconvenience involved in attending acupuncture treatments was seen as a potential barrier, and flexibility in terms of timing and location would be essential. Previous research has confirmed that acupuncture trial participants (including one trial that involved women with PCOS) are willing to attend more frequent treatments (>once a week) if appointment flexibility is offered at the same time [41, 42]. To maximise recruitment and retention rates, we will reduce the duration of twice-weekly appointments to 2 weeks instead of four, and ensure trial acupuncturists are available for appointments outside of business hours.

One of the aims of this study was to determine the impact of pregnancy intention on barriers and facilitators of enrolling in the RCT and potential motivators. Women who were trying to conceive agreed that conception was their highest priority, and that the ideal time to enrol in the trial would be 6 months or more prior to conception. We will use this time frame in our RCT eligibility criteria by excluding women who intend to fall pregnant within the following 6 months. This reflects similar findings from Pastore et al’s RCT on acupuncture for ovulation disorders in PCOS [42]. It was reported that many women were unwilling to cease a health routine to enrol in a study, some women viewed the trial as a limited time opportunity to try something new before commencing fertility treatment, and those seeking pregnancy were less likely to accept randomisation [42]. Therefore, it seems sensible to focus our recruitment efforts on women who are not immediately seeking pregnancy. There was agreement amongst our study participants that weight loss may help facilitate pregnancy and ensure a healthy baby, and this would be a strong motivator for women who were seeking pregnancy in the future. This aligns with expert opinion recommending that overweight or obese women with PCOS make lifestyle modifications prior to commencing fertility treatments [43, 44].

Women in the TTC group suggested the inclusion of peer support groups, which have been shown to have positive effects on self-management of metabolic conditions including PCOS [45–47], previously. However, addition of peer support groups to the RCT is complex and will change the research questions being asked. The role of peer support groups should be explored in future research in women with PCOS, particularly in women who are trying to conceive who may require additional psychological, peer and social support.

### Telephone-based health coaching

We propose using a publicly-funded evidence-based telephone-delivered lifestyle coaching service (GetHealthy NSW) that uses behaviour change/self-regulation principles delivered by a health coach who will assist women to set their own health goals, maintain motivation, and make sustainable changes to diet and physical activity [48]. Women in our study felt that the support and “accountability” of this approach would assist them on their weight loss journey, however some wanted the option to have a more tailored experience with face-to-face individual sessions or more frequent contact if needed. The disadvantages of this approach are a lack of standardisation of the intervention and increased cost. We will not be implementing this in our RCT due to the significant cost associated with individual face-to-face sessions. We chose to use the GetHealthy NSW telephone-based health coaching service because of its affordability, being offered for free to residents of three Australian states, therefore expanding the external validity of our findings. Telephone-delivered lifestyle interventions have the potential to deliver repeated behavioural change interventions in a cost-effective manner [33]. However, incorporating a more tailored experience could be considered in future trials of lifestyle interventions for PCOS - taking a “one size does not fit all” approach.

A perceived disadvantage was that the program is currently not specifically designed for women with PCOS. This could be mitigated by additional training of the health coaches on PCOS. For example, the need for additional psychological support could be highlighted, given the high rates of depression and anxiety symptoms in women with PCOS [49, 50], as well as emphasising the need to demonstrate empathy to this group of vulnerable women. Additional support could be provided by a new PCOS-specific mobile application that is being co-designed in Australia at the National Health and Medical Research Council Centre for Research Excellence in PCOS.

#### Strengths and limitations

It is likely that the study attracted women who have an existing positive attitude towards complementary therapies, therefore inflating the acceptability of our intervention. Consistent with findings from other studies on complementary therapy use [40], the women in our sample were well educated. This highlights the challenge of encouraging the use of a potentially beneficial intervention in a lesser educated cohort who have most to benefit as they have poorer health outcomes.

A potential limitation of this study was the difficulty in recruiting for the focus groups. We had planned for two focus groups of 6–8 participants each. Inclusion of interviews in addition to focus groups allowed us to overcome a pragmatic issue for those women unable to attend a focus group at a specific time, assisting in recruitment of women into this study. Focus groups are useful when gathering data from a group of participants with similar characteristics, to establish when consensus is reached or not between participants. Through the interaction with one another, focus group participants can explore and refine their own ideas, creating a richness in the data [51]. Although group dynamics and iterative development of perceptions through discussion with similar others are not evident in interviews, the consistency in themes identified across interviews and focus groups in this study indicated that similar data was collected regardless of the qualitative method utilised. Additionally, although we did not collect data from as many women as originally planned, saturation of data was reached with no new themes arising towards the end of interviewing.

## Conclusion

Our proposed method for a randomised sham-controlled trial of acupuncture as an adjunct to evidence-based lifestyle interventions appears to be feasible and acceptable to women living in Sydney, Australia. Offering flexibility in timing and location of treatments and modifying the lifestyle intervention to be PCOS-specific, were perceived important for increasing recruitment and retention. The trial is best aimed at those who are not planning to conceive within 6 months. In particular, participants felt there is a clear need for support on the weight loss journey, which ranges from formal and informal social support, support to enable families to change, and facilitation of peer support models. Findings will inform the design of a adequately powered RCT on acupuncture + lifestyle for weight management in overweight or obese women with PCOS.

### Significance

Excess weight significantly worsens metabolic, reproductive and psychological features of PCOS. Compliance to lifestyle interventions is often poor in PCOS with consequent suboptimal weight loss. We have ensured that the interventions proposed in our RCT are feasible, acceptable, useable and replicable, and have been developed from the perspectives of the end user. Should acupuncture be demonstrated in our planned RCT to be an efficacious adjunctive weight loss intervention, this may generate health benefits including improvements in metabolic, reproductive and psychological outcomes.

## Additional files

Additional file 1: Additional Quotes. Additional quotes from the study, not included in the manuscript. (PDF 57 kb)

Additional file 2: Focus group/interview question guide. Question guide used in focus groups/interviews. (PDF 48 kb)

## Abbreviations

BMI: Body mass index
IVF: In vitro fertilisation
OGTT: Oral Glucose Tolerance Test
PCOS: Polycystic ovary syndrome
RCT: Randomised controlled trial
TTC: Trying to conceive
