# Supplementary material for: Feasibility and acceptability of a proposed trial of acupuncture as an adjunct to lifestyle interventions for weight loss in Polycystic Ovary Syndrome: a qualitative study
Source: BMC Complement Altern Med. 2018 Nov 8;18:298. doi: 10.1186/s12906-018-2358-7 (PMC6225727; doi:10.1186/s12906-018-2358-7)
Supplement: Supplementary file 1 — Additional Quotes. Additional quotes from the study, not included in the manuscript. (PDF 57 kb) [file 12906_2018_2358_MOESM1_ESM.pdf]

Additional File 2: Additional quotes

Theme/Subtheme	Example excerpt
**Acupuncture**	
**Advantages**	*"But if it's able to complement those things [diet and exercise] and there's a guide through it I guess, yeah, I think it would be a really good way to do that. Particularly for people with PCOS. Because by the time you probably, certainly for Western culture, if you get to that far you are willing to give anything a go to get the outcome you want. So, depending on what your outcome is. If your outcome is to have a baby and you've done all of it and nothing has happened then I think using that"* - Annabelle, Not TTC<br><br>*" Yeah, I'd be interested to know if that was something that would work. If it was something that I could try then I'd definitely be interested in giving it a go "* - Sarah, TTC<br><br>*"I would love to. I'd say I'd love to try it. If it really helped why not. Like I trust the Chinese medicine "* - Harriet, Not TTC<br><br>*"So, I've started reading about it and there are views and everything. I would say that it's helped some people while some people say it's not. So, I do want to give it a try and I think - but I haven't had an experience yet"* - Keira, TTC<br><br>*"I'd be all for it; I don't see an issue with it. I think it - if it definitely goes towards helping, then I'd definitely be up for it and I wouldn't see why anybody would really have a problem with it"*- Georgia Not TTC
**Experiences with and knowledge of**	*"So, I think it did help with the stress levels really. I think it was more than helping with the cycle. It was more to bring myself down...I thought it was helping me in - because it's something that - it's been around for a long time that's why I was like I'm going to give it a go. So that's my journey with acupuncture"* - Chloe, TTC<br><br>*"Yeah, I used to get it regularly and I found that it helped. It was helping with a few things [headaches, anxiety, dysmenorrhoea]"* Georgia, not TTC<br><br>*"I had electronic acupuncture and I…I can't even remember how it was done now, but I used to absolutely love going...I didn't go for weight management or anything. I went for fertility and I loved going. I felt so relaxed...But after three months there was no results or anything like that so I just had to stop"* - Deanna, TTC

**Disadvantages**	*"Unless you're really into the alternative lifestyle of things and you've delved into that really deeply. It's not something that's mainstream spoken about. So, if you go to your doctor he's not going to say, hey go and have acupuncture"* - Annabelle, not TTC<br>*"But it would still be interesting to see whether you thought you were getting it so you still got the mindfulness"*- Deanna, TTC
**PCOS specific coaching**	*"It's not just the exercise or the [unclear] or the food or the diet. It's part of your life now. It's not a disease we could get a treatment of [unclear], right. It's going to stay with us for the rest of your lives so you need someone - and especially when it comes to support, you need someone who understands that bit"* - unidentified, TTC focus group
**Duration of trial**	*"It's not just for one or two months. It's actually long enough to see whether it does something good about whatever to a certain person, because sometimes you see it's like a month or two months which I do not think it's enough to make a difference to someone"* - Keira, TTC
**Outcomes of importance**	*"I know the link between PCOS and cholesterol and heart disease and stopping or if not preventing as much as possible insulin resistance. Because that's one of the biggest things that will give benefit to that. So that would be all health things I'd be looking at if I can avoid those I'd be very happy"*- Annabelle, TTC

TTC = Trying to conceive
